# The Influence of the Defect Rate of Graphene on Its Reinforcing Capability Within High-Entropy Alloys

**DOI:** 10.3390/nano15151177

**Published:** 2025-07-30

**Authors:** Xianhe Zhang, Hongyun Wang, Chunpei Zhang, Cun Zhang, Xuyao Zhang

**Affiliations:** 1 Hebei Research Center of the Basic Discipline Engineering Mechanics, Shijiazhuang Tiedao University, Shijiazhuang 050043, China; 2 Hebei Key Laboratory of Mechanics of Intelligent Materials and Structures, Shijiazhuang Tiedao University, Shijiazhuang 050043, China; 15531167607@163.com (H.W.); 15630484977@163.com (C.Z.); 3 State Key Laboratory for Turbulence and Complex Systems, Department of Mechanics and Engineering Science, College of Engineering, Peking University, Beijing 100871, China; xyzhang@pku.edu.cn

**Keywords:** graphene, high-entropy alloys, vacancy defects, molecular dynamics, dislocations

## Abstract

Graphene, a remarkable two-dimensional material, enhances the mechanical properties of high-entropy alloys as a reinforcing phase. This study investigated the influence of vacancy defects in graphene on the strengthening effect of FeNiCrCoCu high-entropy alloy through molecular dynamics simulations. The findings reveal that vacancy defects diminish graphene’s strength, resulting in its premature failure. In tensile tests, graphene with defects lowers the yield stress of the composite, yet it retains the ability to impede dislocations. Conversely, graphene exhibits a more pronounced strengthening effect during compression. Specifically, when the deletion of C atoms is less than 1%, the impact is negligible; between 1% and 6%, the strengthening effect diminishes; and when it surpasses 6%, the strengthening effect virtually ceases to exist. This research offers a theoretical foundation for optimizing graphene-reinforced composites.

## 1. Introduction

In recent years, high-entropy alloys (HEAs) have garnered significant attention from researchers worldwide as an innovative metallic material. Numerous experiments have demonstrated that HEAs exhibit exceptional mechanical properties, encompassing superior strength, fracture toughness, creep resistance, and radiation tolerance [1,2,3,4]. A highly illustrative example is the FeNiCrCoCu HEA, which benefits from the significant mixing entropy effect arising from the minimal atomic size differences among its elements [5], making it capable of successfully forming a single-phase FCC solid solution structure [6]. It has recently been established that this alloy is viable and possesses vast application potential. Nevertheless, as engineering application demands continue to escalate, the pivotal challenge lies in further enhancing the strength and toughness of high-entropy alloys while preserving their exceptional performance characteristics. In recent years, numerous studies have been conducted on this type of HEA, with significant efforts made to enhance its mechanical properties. Among these efforts, the method of incorporating reinforcing phases has proven particularly effective. For instance, Zhiming Gao and his team achieved a remarkable synergistic effect through the introduction of Ti and C elements into FeNiCrCo high-entropy alloy, resulting in solid solution strengthening, second phase strengthening, and grain refinement strengthening, thereby significantly enhancing the hardness and wear resistance of the composite material [7]. Ye et al. [8] conducted a study on the reinforcing effect of Gr on CoCrFeNiMn HEA. Their findings revealed that the incorporation of graphite nanosheets led to a 52.99% increase in maximum yield strength and a 28.78% enhancement in ultimate tensile strength. A study has revealed that, thanks to Gr strengthening, the tensile strength of laminated CoCrFeNiMn HEA matrix composites has been enhanced from 673.49 MPa to 826.39 MPa, while its compressive strength has also increased from 2177.47 MPa to 2530.13 MPa [9]. This enhancement is attributed to Gr’s ability to impede the propagation of dislocations, thereby facilitating the strengthening of the composite material. Another factor that boosts strength is the refinement of grain size and the emergence of bulk twins, which similarly hinder the gliding of dislocations. Shi et al. [10] observed that the addition of Gr reinforcement to CoCrFeMnNi HEA results in an interface that serves as a source of non-uniform dislocations. These remarkable mechanical properties, along with the flexibility in their compositional design, offer immense potential for the widespread adoption and utilization of high-entropy alloys.

Graphene (Gr) serves as an exceptional reinforcing phase for enhancing the strength of metal-based materials [11,12]. Zhou et al. presented a cost-effective method to produce oxidation-resistant Cu particles coated with graphene via flash joule heating, exhibiting stable conductivity and effective electromagnetic interference shielding [13]. Li et al. used molecular dynamics simulations to investigate the nano scratching response of HEA/Gr composites, revealing enhanced wear resistance and mechanism transitions due to graphene nanosheet introduction and embedding depth, aiding in the design of composites with superior mechanical and tribological properties [14]. Ye et al. demonstrated an effective strategy for enhancing the tribological performance of HEAs by incorporating a few-layer graphene, resulting in significant reductions in wear rate and friction coefficient, and clarifying the subsurface structure evolution and deformation mechanisms [15]. Nevertheless, its integration into HEA remains scarce, and there is a paucity of research exploring the mechanical properties and Gr reinforcement mechanisms of HEA/Gr composites. Prior studies have simulated the reinforcement mechanism of Gr in HEA by incorporating a monolayer of Gr, revealing that Gr can bolster model strength by inhibiting dislocation nucleation, impeding dislocation movement, and facilitating the formation of stationary dislocations. However, it is noteworthy that Gr prepared experimentally often exhibits a certain degree of defects, necessitating crucial research into the impact of these defects on the strengthening effect of HEA.

Some experimental studies have utilized transmission electron microscopy and scanning tunneling microscopy to corroborate the presence of two distinct types of defects in Gr: inherent natural defects and exogenous defects. Among the natural defects, Stone–Wales defects, vacancy defects, and line defects are included [16,17,18,19,20]. Sethurajaperumal et al. emphasized that the defects present in exfoliated graphene nanosheets are influenced by both the size of the precursor graphite flakes and their inherent imperfections [21]. According to several studies, the presence of vacancy defects can lead to a reduction in the Young’s modules of Gr [22,23,24,25,26]. Robertson and his colleagues induced vacancy defects in graphene through techniques like ion irradiation, and conducted a study on the stability and persistence of certain defects in Gr at the nanoscale, revealing that they are sufficiently stable to persist over time [22]. Pham et al. presented the first atomic-scale investigation of point defects in oxygen plasma-treated epitaxial graphene on SiC, revealing defect-induced states and atomic configurations that may aid in precise defect engineering for metal intercalation [27]. Damasceno et al. [28] employed molecular dynamics and the atomic-scale finite element method (AFEM) in their study and found that the tensile strength properties of graphene exhibit complex dependencies on the shape and size of defects. Sun et al. [29] found through molecular dynamics studies that the fracture strength of graphene decreased by approximately 17.7% due to the presence of a single vacancy. The stress concentration around the vacancy defect leads to the destruction of the nearby hexagonal ring structure, thereby forming an initial crack. Zandiatashbar et al. [30] reported in their experimental study that the mechanical properties of graphene significantly decreased within the range of vacancy defects. In recent years, numerous remarkable achievements have been made in the investigation of Gr vacancy defects. Nevertheless, there has been limited exploration into the impact of these defects on Gr serving as a reinforcing phase in HEA. Given that HEA/Gr composites possess excellent mechanical properties, and the incorporation of Gr as a reinforcing phase significantly enhances the performance of HEA, it is imperative to conduct thorough research on Gr defects. However, it remains challenging to specifically observe the impact of defects in Gr on the overall structure in experiments, necessitating the elucidation of the interaction between Gr and HEA at the atomic level within the nanoscale. Additionally, it is equally difficult to precisely control parameters such as the specific size, quantity, and distribution of defects in Gr during experimental procedures.

To clearly elucidate the impact of vacancy defects in Gr, serving as a reinforcing phase, on the mechanical properties of HEA/Gr composites, as well as the overall microscopic mechanisms, we must explore alternative methods beyond experimentations. Numerous researchers have undertaken studies on Gr-reinforced metallic materials employing molecular dynamics and first-principles methods, yielding significant achievements [31,32]. Fortunately, advances in molecular simulation algorithms and computing capabilities have offered us an alternative avenue to investigate reinforcement mechanisms at the atomic level. For instance, molecular dynamics (MD), a nanoscale computational technique, offers profound insights into atomic-scale observations, which have been extensively utilized to capture underlying mechanisms [33]. The OVITO software enables the visualization of deformation results from MD simulations. The dislocation extraction algorithm (DXA) was employed to detect dislocation features within a nanoscale simulation study of the tensile and compressive FeNiCrCoCu HEA/Gr composite materials using dynamics methods. During the modeling process, multiple vacancy defects eliminated C atoms from Gr, and the impact of these Gr vacancy defects on the strength of the composite model was thoroughly analyzed. The simulation outcomes provide valuable theoretical insights for future molecular dynamics studies and the design of graphene-reinforced metal matrix composites.

## 2. Materials and Methods

### 2.1. Modeling

**Figure 1:**
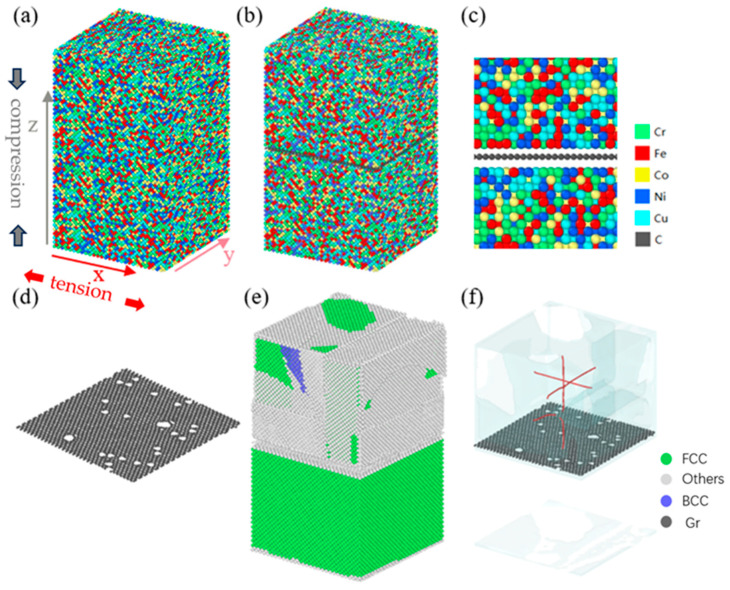
(**a**) Atomic configuration of a pure HEA model; (**b**) atomic configuration of the HEA/Gr model; (**c**) local atomic structure of the HEA matrix and Gr flakes; (**d**) Gr* model with 1% of C atoms removed; (**e**) lattice type of the HEA/Gr* model incorporating edge dislocations; (**f**) defects and dislocation distribution in the HEA/Gr* model with edge dislocations incorporated.

In our current work, all MD simulations were carried out utilizing LAMMPS software [34]. During the model construction process, a single-crystal copper model was initially established. Subsequently, 4/5 of the copper atoms were uniformly replaced with 4 distinct metal atoms to yield a FeNiCrCoCu HEA with identical elemental proportions and a random distribution of each atomic species. The model dimensions were measured to be 10 × 10 × 17 nm, as depicted in Figure 1a. The x, y, and z axes are aligned with the [-110], [-1-12], and [111] directions of the HEA matrix, respectively. Considering that the lattice constant of graphene is about 0.246 nm; to avoid atomic overlapping, it was necessary to reserve 0.246 nm or more in both directions above and below the insertion position of graphene. Therefore, we reserved a gap of 0.5 nm between high-entropy alloys for inserting graphene, and the excess gap was automatically eliminated after the model relaxed, as depicted in Figure 1c. Ultimately, a monolayer Gr sheet, identical in base dimensions, was seamlessly integrated into the HEA model [35]. To eliminate any potential influence stemming from the orientation of Gr in the context of this study, all models of Gr high-entropy alloy composite materials utilized in this simulation work incorporated Gr with a consistent orientation, as depicted in Figure 1d. Consequently, a HEA/Gr composite model was successfully established, with its atomic configuration depicted in Figure 1b. To further explore the impact of Gr on dislocations, we conducted an additional set of simulations for comparison. Specifically, edge dislocations were introduced in the central region of the upper half of the model, aligned with the coordinate system, and extending approximately 22 nm in length. The lattice configuration of the model and the precise location of the introduced dislocation lines are illustrated in Figure 1e,f. After introducing dislocations, the lattice type of the upper section of the model underwent significant alterations, giving rise to unidentifiable lattice patterns on the model’s surface. The dislocations revealed by OVITO software indicate the presence of numerous defective grids at the insertion points of graphene and across the model’s surface. To create multiple vacancy defects, we randomly eliminated C atoms from Gr, relaxing the structure subsequently. The resulting model, depicted in Figure 1d and denoted as Gr*, was generated by removing 1% of the C atoms. Notably, when removing C atoms, if the number of atoms deleted is even, the C atoms can be fully reconstructed, thus avoiding the formation of dangling bonds. However, the absence of an odd number of atoms can render Gr more unstable and reactive due to the presence of dangling bonds [36].

### 2.2. Interatomic Potentials

In this study, we utilized LAMMPS to simulate the aforementioned model, employing the embedded atom method (EAM) potential to delineate the intricate interactions among metal atoms. The EAM potential function model comprises both atomic and environmental attributes, effectively simulating the interactions between atoms as well as the structural behavior of atoms within crystals. This approach exhibits remarkable accuracy in characterizing the metal structure, mechanical properties, and thermodynamic behaviors of real-world materials. Specifically, the EAM potential comprises a pair potential and an embedding energy and is expressed as follows:

(1) $$u_{EAM}=\sum_{i=1}^{N}\sum_{j=i+1}^{N}u\left(r_{ij}\right)+\sum_{i=1}^{N}E_{i}\left(\rho_{i}\right)$$

In the equation, *U_EAM_* represents the total potential energy of the entire system, *r_ij_* denotes the distance between atom *i* and atom *j*, *E_i_* signifies the embedding energy of atom *i*, and *ρ_i_* indicates the atom *i* by all other atoms within the system.

To be specific, this formula can be formulated as follows:

(2) $$\rho_{i}=\sum_{j=1,j\neq i}^{N}\rho_{j}\left(r_{ij}\right)$$

In the equation, *ρ_j_*(*r_ij_*) represents the electron density induced by atom *j* at position of atom *i*.

For FeNiCrCoCu high-entropy alloys, the EAM potential reported by Farkas and Caro [37] characterizes the interactions among Fe, Ni, Cr, Co, and Cu atoms, which has been validated in prior investigations pertaining to the FeNiCrCoCu HEA system [38,39,40]. Using empirical potentials, the average lattice constant [41] and elastic constant [42] of FeNiCrCoCu HEA have been measured to be highly comparable to both experimental values and density functional theory calculations [43].

The AIREBO potential [44] is employed to characterize the C-C bond interactions within Gr flakes, and this potential function has been extensively utilized in the investigation of carbon nanomaterials. Additionally, given that the interaction between metals and carbon is significantly weaker compared to that among metal atoms, the application of the Lennard–Jones (L-J) interaction potential suffices to furnish a reasonable approximation for the interaction between metals and carbon atoms [45,46,47,48]. It has been extensively utilized in a diverse array of composite systems to characterize metal–Gr interactions [49,50,51,52]. Consequently, the van der Waals force interaction between HEA and Gr can be accurately captured by a prototypical L-J potential [40], with the specific form of force action outlined as follows:

(3) $$V_{LJ}(r)=4\varepsilon \left[{\left(\frac{\sigma }{r}\right)}^{12}-{\left(\frac{\sigma }{r}\right)}^{6}\right]$$

In the equation, *r* represents the distance between two particles, *σ* represents the critical distance at which the potential energy is zero, and *ε* signifies the depth of the potential energy function.

The L-J potential energy function comprises two components: the attraction term, which characterizes the van der Waals attraction among atoms, and the repulsion term, which accounts for the van der Waals repulsion between atoms. Together, these two terms determine the overall energy of atomic interactions. The L-J force field, a type of non-bonding force field, is employed to describe the interactions of non-covalent bonds, including van der Waals forces and electrostatic interactions, within molecules. The parameters of the L-J force field can be derived through experimental measurements or computational methods and can be further adjusted and optimized based on the specific simulated system. The values of σ and ε for various elements are detailed in Table 1.

### 2.3. Simulations

During the simulation, all three boundaries were periodic, and the entire process was categorized into two distinct stages: relaxation and loading. Prior to loading the model, the conjugate gradient method was employed to minimize the model’s energy, utilizing a step size of 0.001 ps.

We performed time integration using Nose–Hoover non-Hamiltonian equations to sample particle positions and velocities from canonical (NVT) and isothermal–isobaric (NPT) ensembles. The NVT ensemble maintains a constant particle number, volume, and temperature while allowing energy fluctuations, making it suitable for volume-sensitive studies where pressure effects must be minimized. The NPT ensemble also maintains the particle number, pressure, and temperature, enabling accurate simulation of materials’ mechanical behavior under realistic stress conditions. Additionally, the Langevin method introduces random and damping forces to simulate thermal baths, proving particularly effective for complex systems, like tribological interfaces and heat transfer scenarios, where conventional thermal baths may fail. In this study, we employed the NPT approach during the isothermal relaxation and mechanical loading of high-entropy alloy/graphene composites to replicate their experimental deformation under stress. This methodology allows for precise control over thermodynamic conditions while observing material responses, bridging the gap between computational models and physical experiments. Temperature control was achieved through the utilization of NVT and NPT ensembles. Given the diminished thermal stability of Gr, once it reached 800 K, we established 800 K as the maximum relaxation temperature to prevent any potential damage to Gr during the relaxation phase. Subsequently, the temperature was maintained at a constant 300 K throughout the loading process. During the relaxation process, the temperature was initially raised from 300 K to 800 K using the NPT ensemble within 100 ps, followed by maintaining a constant temperature of 800 K for 50 ps with the NVT ensemble. Subsequently, the NPT ensemble was employed for the cooling phase, gradually reducing the temperature from 800 K back to 300 K after 100 ps. The model was then maintained at a constant temperature of 300 K for an additional 50 ps. This heating and cooling relaxation procedure allowed the model to effectively release internal stress. Upon completion of relaxation, loading was initiated. Two distinct loading modes were employed in this study: stretching along the x-axis and compression along the z-axis. The time step of the molecular dynamics simulation was set at the femtosecond (fs, 10^−15^ s) level. Accordingly, the commonly used strain rate range for molecular dynamics simulations of ductile materials is 10^8^~10^11^ s^−1^ (corresponding to a tensile rate of 0.001~1 ps^−1^). The strain rate used in our simulation was 10^9^ s^−1^, which is commonly used in molecular dynamics simulations of mechanical deformation of metallic materials [54,55]. All model simulations presented in this article adhered to the relaxation and loading procedures outlined above. In practical preparation, the sintering temperature of chromium carbide may approximate 1273 K [15]. Given that the maximum processing temperature in this study was 800 K, the impact of carbide chemical reactions on the simulation results can be disregarded.

## 3. Results

### 3.1. The Impact of Gr Vacancy Defects on the Tensile Performance

**Figure 2:**
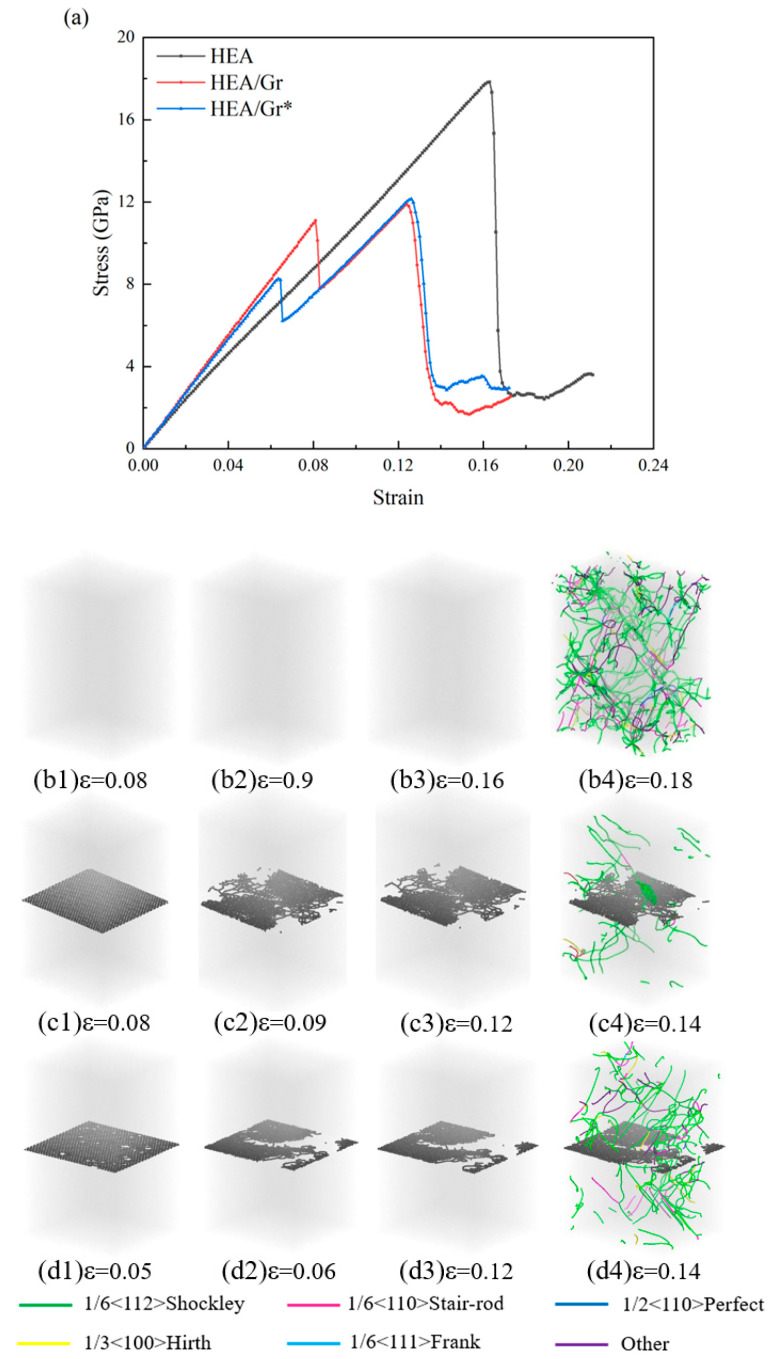
Tensile stress–strain curves of pure HEA, HEA/Gr, and HEA/Gr* (with 1% of C atoms removed in Gr) models without the introduction of initial dislocations (**a**); dislocation distribution of pure HEA (**b1**–**b4**), HEA/Gr (**c1**–**c4**), and HEA/Gr (**d1**–**d4**) under various tensile strains.

Figure 2 presents the stress–strain curves and dislocation distributions across various strain levels for three distinct models: pure HEA, HEA/Gr, and HEA/Gr*. Notably, all these datasets were derived from tensile simulations conducted along the x-axis, without the introduction of initial dislocations. Specifically, HEA/Gr* denotes a modified version of the model, where 1% of the C atoms in Gr have been removed. Prior investigations have thoroughly analyzed and drawn conclusions from the data obtained from both the HEA and HEA/Gr models. However, due to the premature failure of Gr, unexpected defects emerged in the single-crystal HEA, ultimately leading to a significantly lower yield strength in the HEA/Gr model compared to the pure HEA model. After introducing defects into Gr, similar outcomes were observed. As depicted in Figure 2a, during the initial stages of strain, the high modulus of Gr contributed to an enhancement in the overall elastic modulus. Notably, the curves of the two models incorporating Gr exhibited a close resemblance, albeit with the elastic modulus of HEA/Gr* being slightly inferior to that of HEA/Gr. Examining the dislocation distribution depicted in Figure 2, it becomes evident that during the subsequent stretching process, the failure of Gr preceded the failure of HEA, resulting in the formation of numerous defects within the model. These defects provided fertile ground for dislocation nucleation, manifesting as a downward shift in stress within the stress–strain curve. Notably, even though HEA retains its load-bearing capacity during this phase, the HEA/Gr model exhibited a lower yield stress compared to the pure HEA model. Furthermore, in the case of the HEA/Gr* model, the presence of vacancy defects within Gr rendered it more susceptible to failure. Consequently, Figure 2a illustrates an earlier occurrence of stress mutation in Gr. However, given the similarity in the state of HEA, the yield stress and yield strain of the HEA/Gr* model remained closely aligned with those of the HEA/Gr model. Nevertheless, a noteworthy observation is that the HEA/Gr* model exhibited a higher flow stress following yield. As evident from (c4) and (d4) in Figure 2, the model exhibited a significant generation of dislocations following yielding. Notably, the quantity of dislocations observed in (d4) is conspicuously greater than that in (c4). This disparity can be attributed to the inherent vacancy defects in Gr*, which contributed to the formation of a higher number of defects upon failure. Consequently, during the overall yielding process of the model, a greater number of dislocations nucleated, leading to the emergence of numerous dislocation lines. Among these dislocation lines, green Shockley dislocations and stair-rod dislocations constituted the largest proportion. Notably, stair-rod dislocations possessed the lowest dislocation energy among all types, indicating its ability to counteract substantial stress. Therefore, it is widely recognized that stair-rod dislocation plays a pivotal role in enhancing the strength of the matrix. An increase in the number of dislocations enhanced the strength of the matrix, evident from the comparison of stress–strain curves between the HEA/Gr and HEA/Gr* models, where the flow stress after yielding was notably higher in the HEA/Gr* model than in the HEA/Gr model.

**Figure 3:**
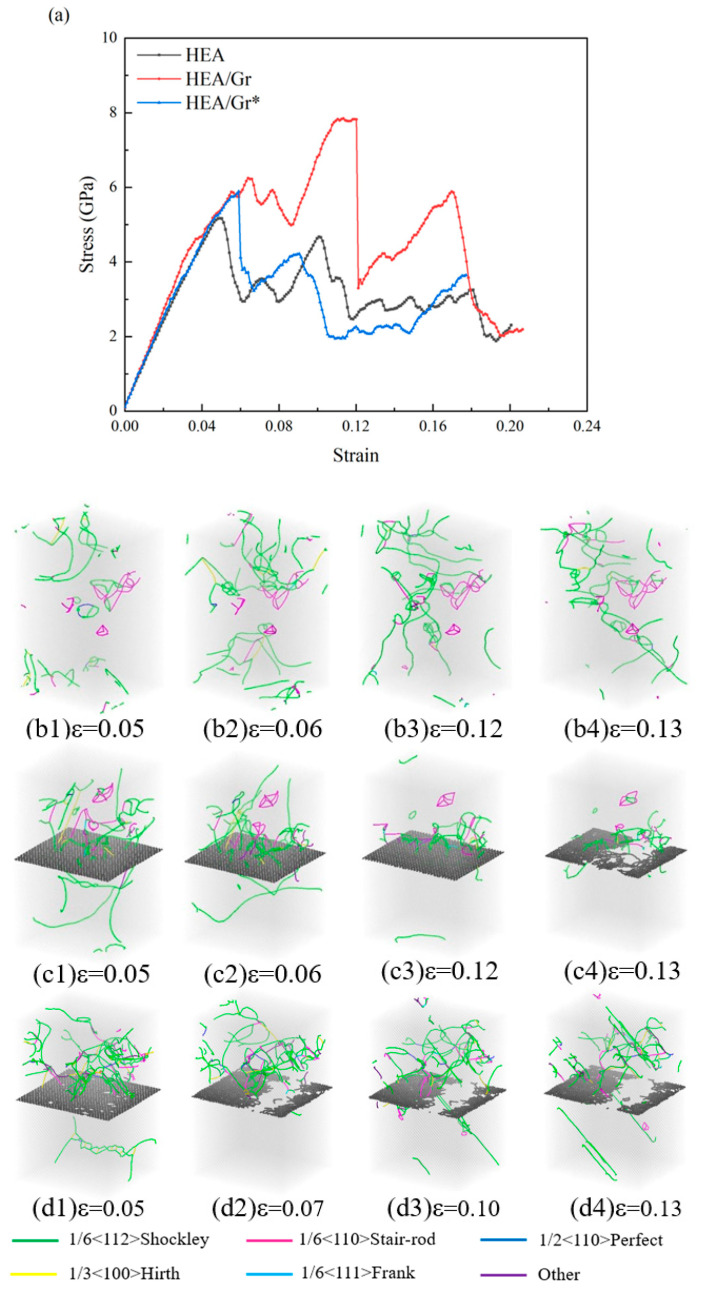
Tensile stress–strain curves of pure HEA, HEA/Gr, and HEA/Gr* (with 1% of C atoms removed in Gr) models incorporating initial dislocation (**a**); dislocation distribution of pure HEA (**b1**–**b4**), HEA/Gr (**c1**–**c4**), and HEA/Gr (**d1**–**d4**) under varying tensile strains.

The stress–strain data and dislocation distribution across various strain levels, obtained through the tensile simulation of the model incorporating initial dislocations along the x-axis, are depicted in Figure 3. Upon examination of Figure 3a, it becomes evident that the strength of all three models underwent a notable decline. When a certain number of initial dislocations were added to the upper half of the HEA/Gr model, simulation results show that the yield strength and toughness of both models significantly decreased, with little effect on the flow stress after yielding. The process can be divided into five different strain stages: elastic stage, local yield stage, secondary elastic stage, overall yield stage, and plastic stage. Due to the introduction of dislocations, the upper alloy yielded, while graphene hindered the propagation of dislocations, resulting in the lower part remaining elastic, thus leading to a secondary elastic stage. Notably, the curves within the elastic phase exhibited remarkable similarity, with a narrowed gap in the elastic modulus. This observation suggests a diminished role of Gr in load-bearing capabilities, with its primary influence being the modulation of the model’s initial dislocations. Prior to entering the local yielding stage, specifically during the initial yielding of the HEA/Gr model, the stress levels exhibited by both the HEA/Gr and HEA/Gr* models were approximately equivalent and significantly surpassed that of the pure HEA model. Upon the local yielding of HEA, Gr assumed the role of load bearing. However, at this juncture, Gr* with vacancy defects experienced rapid failure, rendering it incapable of enduring further stress. Consequently, the subsequent curve trend of the model closely resembles that of the pure HEA, lacking any subsequent secondary elasticity or overall yielding stages. From a comparative analysis of (b1–b3) and (c1–c3) in Figure 3, it becomes evident that Gr served as an effective barrier, significantly impeding the migration of initial dislocations from the upper section of the model towards the region beneath Gr. This hindrance mitigates the detrimental impact of initial dislocations on the overall structural integrity of the model, thereby enhancing its overall yield strength. Furthermore, Figure 2d1 illustrates that despite the presence of vacancy defects in Gr, it retained a certain degree of resistance against dislocations. Consequently, only a minimal number of dislocation lines were formed in the lower portion. However, these vacancies compromised the strength of Gr, leading to its fracture, as depicted in Figure 3d2. Subsequently, the overall strength experienced a sudden downward shift, resulting in a rapid yield.

**Figure 4:**
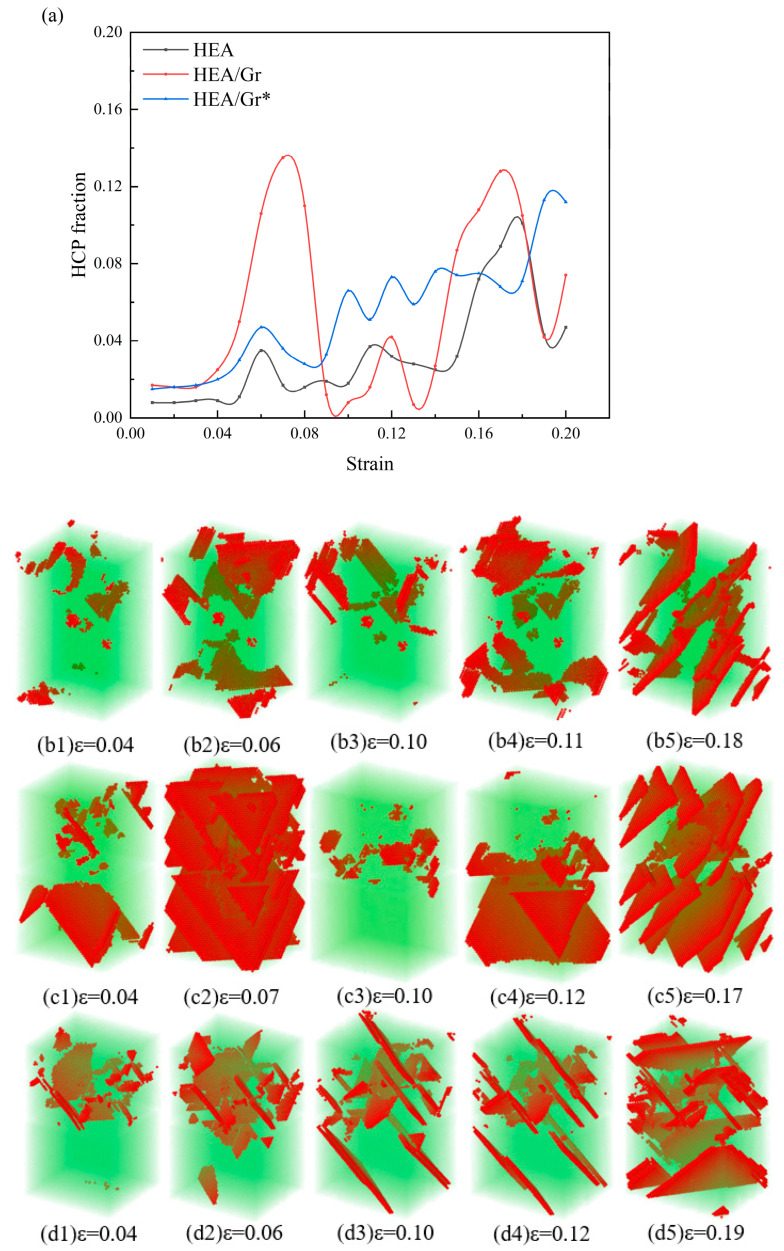
Evolution of the HCP fraction during the tensile process of pure HEA, HEA/Gr, and HEA/Gr* (with 1% of C atoms removed in Gr) models in the presence of initial dislocations (**a**); twinning variations observed in pure HEA (**b1**–**b5**), HEA/Gr (**c1**–**c5**), and HEA/Gr (**d1**–**d5**).

In Figure 4a, the variations in hexagonal close-packed (HCP) scores across the models are evident. These changes exhibit a remarkable similarity among the models, with the numerical ranking being HEA/Gr > HEA/Gr* > pure HEA. This alignment coincides with the descending order of their respective strengths. This concordance can be attributed to the fact that the number of HCPs influences the degree of difficulty in model deformation; specifically, a higher count of HCPs renders the model less susceptible to deformation. A comparative analysis of the HEA/Gr curves in Figure 3a and Figure 4a reveals a pattern: abrupt changes in stress are frequently accompanied by corresponding shifts in HCP scores. A similar trend is observed in HEA/Gr*, albeit with a narrower range of variation and a more undulating curve. Comparing the twinning variations among the three models depicted in Figure 4, it becomes evident that only HEA/Gr exhibited a significant generation of twins in both the upper and lower sections of the model. In contrast, pure HEA and HEA/Gr* primarily exhibited twinning in the upper portion, with only a minor presence in the lower portion. Prior to the failure of Gr, the twinning directions varied among the three models. This variance can be attributed to the fact that once the HEA model entered the local yield stage in the upper section, its lower section remained capable of bearing the load. Consequently, a contraction force, opposing the tensile direction, arose at the junction between the upper and lower parts, giving rise to the twinning direction observed in Figure 4b2. For the HEA/Gr model, Gr’s central position and superior strength enabled it to exert a contraction force that extended to the lower half of the model. Consequently, this resulted in the twinning transformation depicted in Figure 4c2, where numerous twins were formed in both the upper and lower sections due to the contraction force. Once the model reached its overall yield point, it ceased to generate a reverse contraction force, leading to the formation of twins, as observed in Figure 4b5,c5. On the other hand, in the HEA/Gr* model, the presence of vacancy defects weakened the strength of Gr, preventing it from exerting a contraction force that affected both halves of the model. Instead, similar to pure HEA, a contraction force was generated at the interface between HEA and Gr, primarily affecting the upper part of the model, while the lower part could still sustain the load. Consequently, twinning was primarily concentrated in the upper section, and the twinning direction exhibited significant differences from the subsequent stages.

It can be concluded that, during tensile simulations conducted parallel to the graphene direction, Gr* exhibited a hindering effect on dislocations even when containing a certain number of vacancy defects. By limiting the diffusion of dislocation defects, it prevented initial dislocations from causing structural damage, thereby exerting a certain strengthening effect on the model. Nevertheless, the presence of vacancy defects compromised the inherent strength of Gr*, leading to earlier fracture and a reduced number of twins generated. Consequently, the strengthening effect was attenuated compared to Gr without vacancy defects.

### 3.2. The Impact of Gr Vacancy Defects on the Compressive Performance

**Figure 5:**
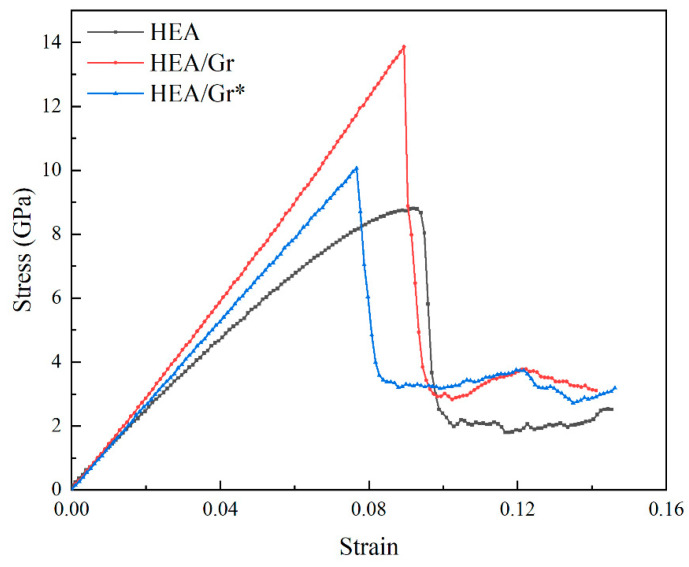
Compressive stress–strain curves of pure HEA, HEA/Gr, and HEA/Gr* (with 1% of C atoms removed in Gr*) models, without the introduction of initial dislocations.

**Figure 6:**
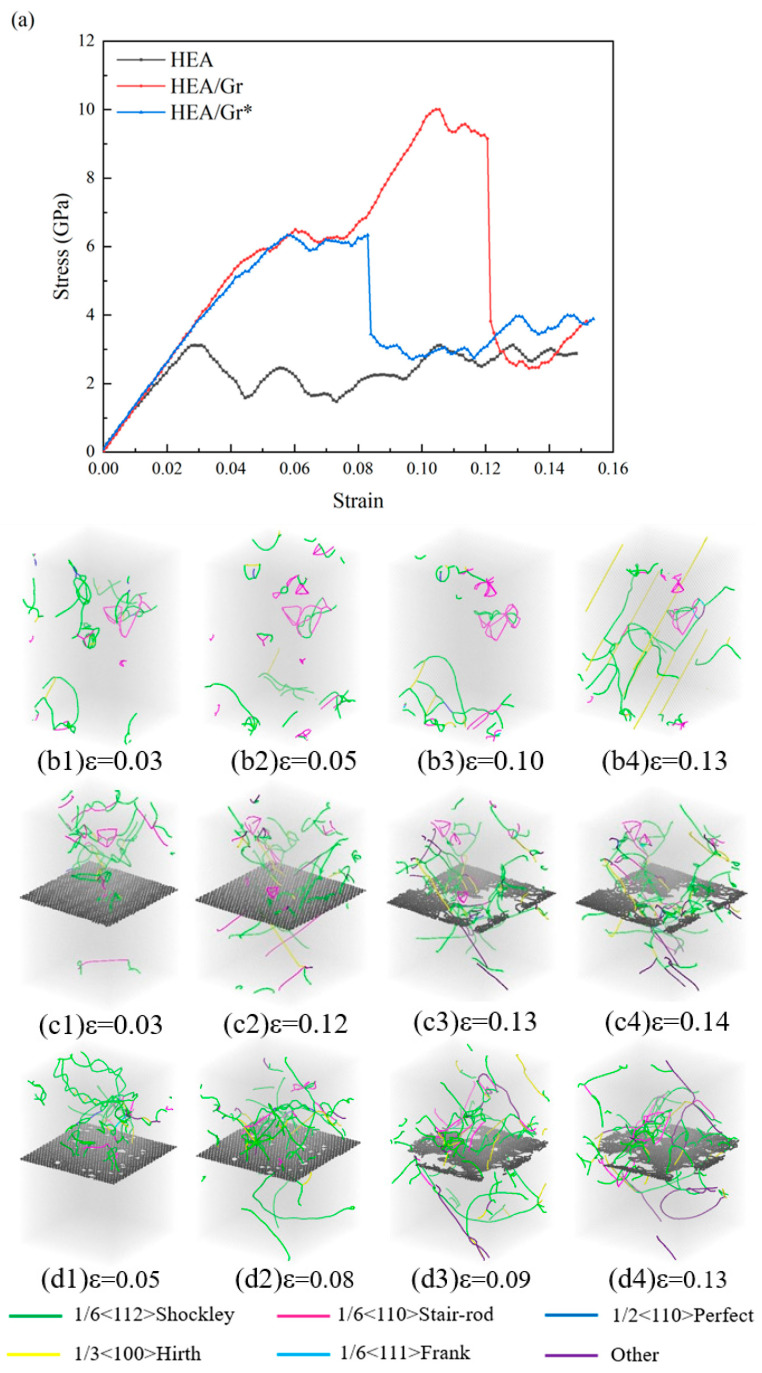
Compressive stress–strain curves of pure HEA, HEA/Gr, and HEA/Gr* (with 1% of C atoms removed in Gr) models incorporating initial dislocations (**a**); dislocation distribution of pure HEA (**b1**–**b4**), HEA/Gr (**c1**–**c4**), and HEA/Gr (**d1**–**d4**) under varying compressive strains.

Compared to tensile simulations, Gr exhibited more pronounced strengthening when the model was subjected to pressure, and the influence of vacancy defects on Gr* strengthening was also more evident. The model utilized in this simulation is identical to the one employed in the preceding section. Figure 5 illustrates the stress–strain curve under compression along the z-axis. Notably, during the elastic stage, there were distinct disparities in the elastic modulus and yield strength among the three models. Specifically, the HEA/Gr model exhibited the highest elastic modulus and yield strength. However, the presence of vacancy defects in Gr* significantly diminished the elastic modulus and yield strength of the HEA/Gr* model, albeit still remaining significantly superior to those of the pure HEA model. Concurrently, these vacancy defects compromised the load bearing and deformation capabilities of Gr. Consequently, due to the failure of Gr, the HEA/Gr* model was the first to yield among the three simulations. In fact, the strengthening mechanism of Gr on the matrix model primarily relied on its influence on dislocations. For models devoid of initial dislocations, there is no nucleation of dislocations prior to yielding. Consequently, the enhancement of the yield strength of the model by Gr is primarily attributed to its inherent high compressive strength. However, once the model yielded, a significant number of dislocations nucleated, and Gr effectively hindered these dislocations, thereby strengthening the flow stress of the model. As evident from the plastic stage depicted in Figure 5 and Figure 6, the flow stress of both the HEA/Gr and HEA/Gr* models is comparable and superior to that of the pure HEA model. This underscores the fact that, despite reducing the strength of Gr* itself, vacancy defects do not impede its ability to affect dislocations and consequently strengthen the model by hindering their movement.

After incorporating initial dislocations, the stress–strain curves and dislocation distributions resulting from the compression simulation of the three models are presented in Figure 6. It was established that Gr primarily enhanced the matrix strength by influencing dislocations, and its impact was even more pronounced in the presence of initial dislocations. According to the stress–strain curves depicted in Figure 6a, the yield stress of both models incorporating Gr exceeded that of the pure HEA model. Notably, the* model retained a high strength level during the initial yield, comparable to the HEA/Gr model, suggesting that despite having vacancy defects, Gr* can effectively hinder the propagation of initial dislocations. Furthermore, the dislocation distribution shown in Figure 6b1,d1 reveals that the model containing Gr* exhibited a significantly reduced number of dislocations in its lower half, whereas the initial dislocations in the pure HEA model diffused into other regions. After experiencing stress oscillation and entering the second elastic stage, Gr* was unable to sustain further stress and was promptly crushed, leading to the overall transition into the yield stage. Conversely, in the HEA/Gr model, the absence of vacancy defects allowed Gr to endure greater forces, thereby enhancing the overall model strength.

In summary, it can be deduced that Gr plays a more significant role in the compression simulation conducted perpendicular to the graphene surface, while vacancy defects exert a greater influence on the strengthening effect of Gr. Despite the presence of dislocation defects, Gr can still impede dislocations, exerting a certain strengthening impact on the model during the initial stages of strain. Furthermore, it continues to enhance flow stress even after the overall yield of the model. Nevertheless, the inherent strength of Gr* is rather low, rendering it susceptible to rapid damage when subjected to loading. Consequently, its strengthening effect is attenuated compared to Gr without vacancy defects.

### 3.3. The Impact of Varying Quantities of Vacancy Defects on the Enhancement Effect

**Figure 7:**
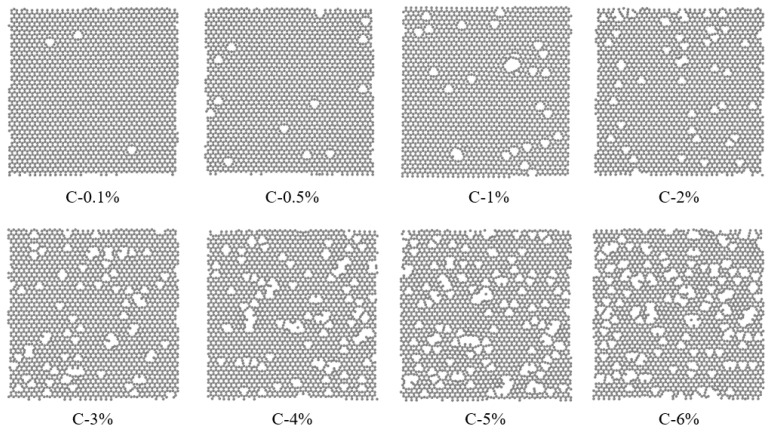
Model after relaxation of Gr with varying quantities of C atoms removed.

After discussing the distinct strengthening effects of Gr and Gr* on the model, we further investigated the impact of varying vacancy defect concentrations on the strengthening capabilities of Gr by meticulously controlling the number of randomly deleted C atoms. Based on our prior discussions, it is evident that the primary mechanism of Gr’s strengthening effect lies in its ability to hinder dislocations. Consequently, in this section, we introduced initial dislocations to the upper portion of the model, ensuring that the number and location of these dislocations remained consistent across all models, with the sole exception being the varying number of C atoms within Gr. Figure 7 presents the relaxed Gr models obtained after randomly deleting different percentages of C atoms, specifically 0.1%, 0.5%, 1%, 2%, 3%, 4%, 5%, and 6%. Notably, in the preceding the Gr* utilized HEA/Gr* composite had a C atom deletion rate of 1%. Moreover, the size and orientation of Gr were consistent with the model established above.

**Figure 8:**
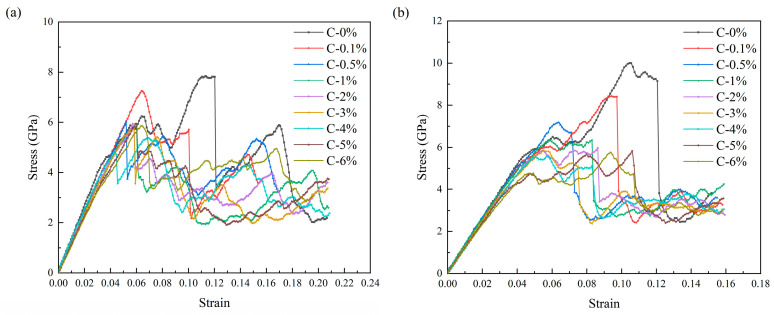
The (**a**) tensile and (**b**) compressive stress–strain curves obtained for HEA/Gr* and HEA/Gr (C-0%) models with C atoms removed at concentrations of 0.1%, 0.5%, 1%, 2%, 3%, 4%, 5%, and 6%, respectively, following the introduction of initial dislocations.

Tensile simulations along the x-direction and compressive simulations along the y-direction were conducted on the HEA/Gr* models and the HEA/Gr models without vacancy defects, respectively, which were composed of the aforementioned eight sets of models. The stress–strain curves obtained are depicted in Figure 8. The number of C atoms in Gr totals 2640, with the number of atoms deleted rounded off to the nearest whole number according to the rule of four. Notably, the model with the minimal defect count involved the deletion of merely 3 C atoms (C-0.1%), whereas the model with the highest defect count saw the removal of 158 C atoms (C-6%). Evidently, Figure 8 illustrates that vacancy defects exerted an influence on the overall strength of the models throughout various stages of strain. This impact became increasingly significant with a rise in the number of defects. Figure 8a depicts the stress–strain curve obtained from the tensile simulation. Evidently, the models incorporating vacancy defects exhibited inferior strength compared to the HEA/Gr model. Notably, in models where the deletion of C atoms exceeded or equaled 0.5%, a downward stress anomaly was observed during the elastic phase. This anomaly arose due to the proliferation of vacancy defects, which diminished the strength of Gr* and precipitated its premature failure. Since Gr’s reinforcement mechanism primarily relies on its inherent strength and the impediment it poses to dislocations, the absence of Gr* following its failure negates its load-bearing capacity. Consequently, dislocations were able to traverse the failed region of Gr*, thereby not only diminishing the yield stress of the model but also advancing the overall yield strain.

**Figure 9:**
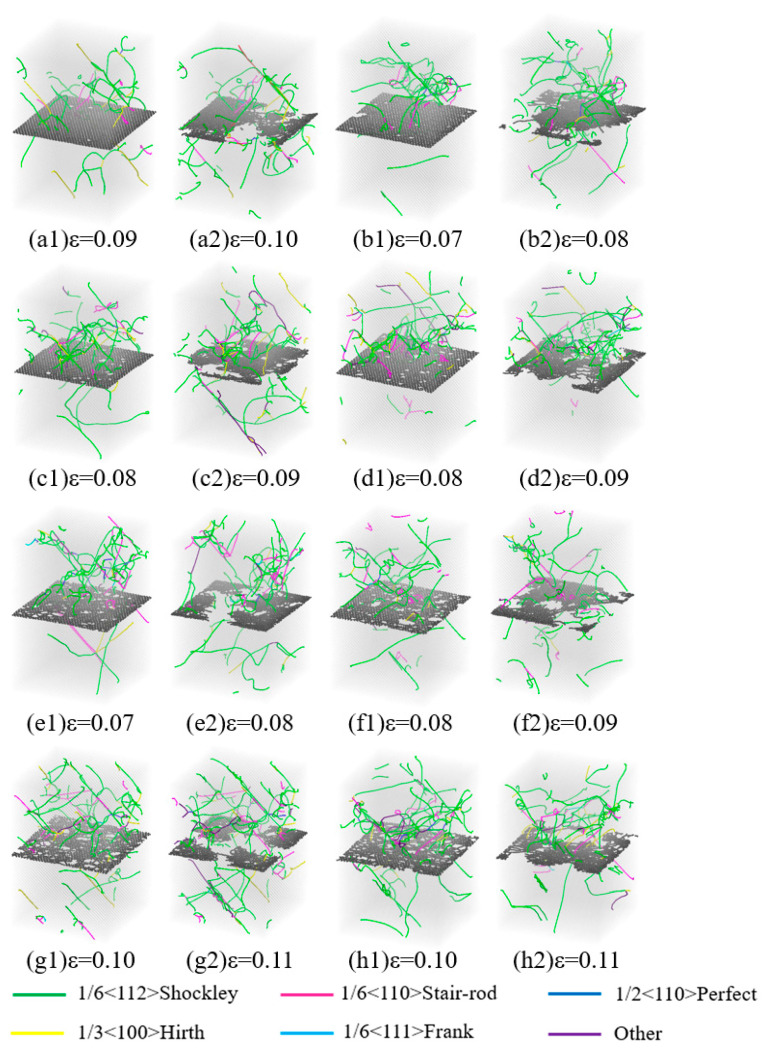
Illustration of the dislocation distribution in the HEA/Gr* models prior to and following Gr* failure during compression simulations, considering various percentages of deleted C atoms ranging from 0.1% to 6% (**a**–**h**), with the inclusion of initial dislocations.

Figure 8b depicts the stress–strain curve obtained from the compression simulation, allowing for similar inferences as drawn from the tensile simulation. Nevertheless, due to the variance in the direction of applied force, Gr* exhibited remarkable compressive resilience under perpendicular pressure loads to its plane, enabling it to sustain greater strains. Notably, Gr* failed only after the yield point of HEA was reached, contrasting with observations from the tensile simulation. Consequently, our analysis primarily focused on elucidating the failure mechanisms and dislocation distributions of Gr* during the compression simulation. Figure 9 illustrates the atomic views of Gr* with varying defect concentrations, both prior to and following its failure, along with the corresponding dislocation patterns under specific strain conditions during the compression simulation. Evidently, the failure mode of Gr* was significantly influenced by defects, with the size and distribution of vacancy defects exerting a profound impact on its deformation behavior. This is attributed to the generation of elevated local stresses at the vacancy defects, which primarily accounted for the reduction in Gr* strength. Figure 9 further reveals that upon the destruction of Gr, dislocations initiated their passage through the damaged area, whereas the unaffected regions continued to impede their movement. Notably, even after the overall yield of the model, Gr retained its capacity to enhance the flow stress. At this juncture, the disparity in defect counts exerted minimal influence, as the flow stress exhibited by HEA/Gr* incorporating various vacancy defects remained comparable to that of the defect-free variant and surpassed the pure HEA model. Prior to the destruction of Gr, the lower section of the model witnessed the emergence of only a limited number of dislocations. However, an increase in defect concentration significantly impacted the dislocation density in this region. A closer inspection of (a1), (b1), (g1), and (h1) in Figure 9 offers a clearer picture of this phenomenon. As the number of vacancy defects escalated, dislocations began to penetrate Gr in smaller quantities, ultimately contributing to the overall reduction in the model’s strength. Contrary to yield strength, the yield strain of the model did not consistently decrease with the augmentation of defects. In fact, when the number of vacancy defects was relatively small, a concentrated distribution of a few defects was more prone to causing damage to the model, whereas a larger but uniformly distributed number of vacancy defects tended to stabilize the material structure and enhance its toughness. Consequently, as shown in Figure 9g,h, which depict the removal of two sets of models containing 5% and 6% C atoms, the strain at Gr* failure was observed to be higher than that of the HEA/Gr* models with varying defect concentrations. This noteworthy trend was also evident in the tensile simulations.

**Figure 10:**
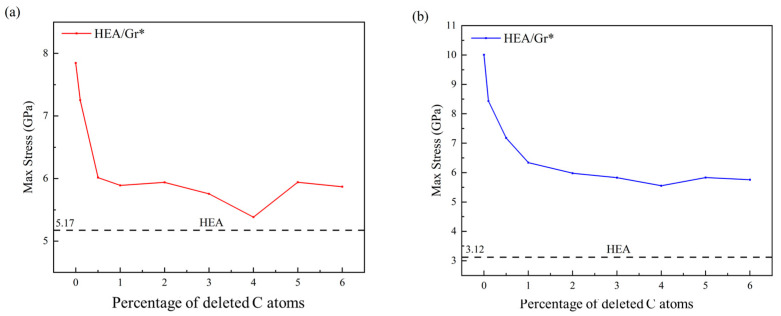
The trends in (**a**) maximum tensile stress and (**b**) maximum compressive stress for the HEA/Gr* and HEA/Gr (C-0%) models, with varying percentages of atoms removed (0.1%, 0.5%, 1%, 2%, 3%, 4%, 5%, and 6%) following the introduction of initial dislocations. The dashed line serves as a reference, depicting the maximum stress values simulated for the pure HEA model under identical conditions.

When the quantity of deleted C atoms surpassed 6% (C > 6%), Gr experienced an excessive number of vacancy defects, greatly compromising its structural stability. Consequently, the enhancement effect on the model approached zero, and its overall strength approximated that of the pure HEA model. Conversely, when the deletion of C atoms remained below 1% (C < 1%), Gr exhibited minimal vacancy defects, rendering the model nearly flawless. In this scenario, Gr* retained a notable enhancement effect on the model. Figure 10 illustrates the trend in maximum stress values across various model groups, with the dashed line representing the maximum stress value simulated by the pure HEA model under identical conditions. Notably, Figure 10 reveals that as the percentage of deleted C atoms ranged from 1% to 6% (1% ≤ C* ≤ 6%), the model’s strength stabilized gradually. Interestingly, when precisely 4% of C atoms were removed, the model’s strength lagged behind all other HEA/Gr* configurations. This anomaly arose from the formation of larger, more clustered vacancies and dangling bonds within the model during the random deletion of 4% C atoms, ultimately reducing the strength of Gr* compared to other groups. Furthermore, it is evident that Gr* exhibited a more pronounced impact on enhancing the model’s compressive strength when confronted with dislocations.

## 4. Conclusions

In summary, we employed molecular dynamics simulations to investigate the impact of vacancy defects on the strengthening effect of Gr in FeNiCrCoCu HEA/Gr systems. Graphene sheets containing varying numbers of vacancy defects were incorporated into a pristine single-crystal high-entropy alloy model. Additionally, initial edge dislocations were introduced in the upper section of the model. Tensile and compressive simulations were performed on diverse HEA/Gr models, which were subsequently compared with HEA/Gr configurations devoid of vacancy defects and pure HEA models. The key findings derived from the comprehensive data analysis are outlined as follows.

(1) Vacancy defects can lead to a reduction in the strength of Gr, rendering it more susceptible to damage when it is required to bear loads. Compared to Gr without such defects, the strengthening effect is diminished. During simulations, the strain nodes associated with Gr failure occur prematurely, yet they still contribute to a certain degree of strengthening for the overall model. Research indicates that Gr* with a minimal amount of vacancy defects remains effective in blocking the movement of dislocations, altering the direction of dislocation propagation and increasing dislocation density. When confronted with initial dislocations, this can enhance the local yield stress of the model. Notably, in compression simulations conducted perpendicular to the graphene plane, Gr* plays a pivotal role in bolstering the flow stress of the model, even after the overall yield point has been reached.
(2) Introducing varying quantities of vacancy defects into Gr* exerts diverse degrees of influence on its strengthening effect. Both the size and distribution of these defects play a pivotal role in shaping the deformation behavior of Gr*. A small yet concentrated number of vacancy defects renders the model more susceptible to damage, whereas a larger quantity of defects distributed uniformly enhances the stability and toughness of the model. When the concentration of vacancy defects in Gr* is low (C*>6%), Gr* struggles to maintain the stability of its structure, suffering damage during the relaxation phase. Consequently, the strengthening effect disappears in the elastic stage, and the model’s overall yield strength approaches that of pure HEA.

## Figures and Tables

**Table 1:** L-J potential parameters between internal atoms of HEA and Gr layers [53].

Pair	σ(Å)	ε(eV)
Fe–C	3.1000	0.050000
Ni–C	2.8520	0.230000
Cr–C	2.8680	0.037758
Co–C	2.8420	0.038281
Cu–C	3.0825	0.025780

## Data Availability

The original contributions presented in the study are included in the article; further inquiries can be directed to the corresponding author.
